# Cause and predictors of neonatal mortality among neonates admitted to neonatal intensive care units of public hospitals in eastern Ethiopia: a facility-based prospective follow-up study

**DOI:** 10.1186/s12887-020-02051-7

**Published:** 2020-04-14

**Authors:** Assefa Desalew, Yitagesu Sintayehu, Nardos Teferi, Firehiwot Amare, Bifitu Geda, Teshager Worku, Kebebush Abera, Abiyot Asefaw

**Affiliations:** 1 School of Nursing and Midwifery, College of Health and Medical Sciences, Haramaya University, Po. Box 235, Harar, Ethiopia; 2 School of Medicine, College of Health and Medical Sciences, Haramaya University, Harar, Ethiopia; 3 School of Pharmacy, College of Health and Medical Sciences, Haramaya University, Harar, Ethiopia

**Keywords:** Facility-based study, Neonatal mortality, Predictors, NICU, Ethiopia

## Abstract

**Background:**

The first month is the most crucial period for child survival. Neonatal mortality continues to remain high with little improvement over the years in Sub-Saharan Africa, including Ethiopia. This region shows the least progress in reducing neonatal mortality and continues to be a significant public health issue. In this study setting, the causes and predictors of neonatal death in the neonatal intensive care units are not well documented. Hence, this study aimed to determine the causes and predictors of neonatal mortality among infants admitted to neonatal intensive care units in eastern Ethiopia.

**Methods:**

A facility-based in prospective follow-up study was conducted among neonates admitted to neonatal intensive care units of public hospitals of eastern Ethiopia from November 1 to December 30, 2018. Data were collected using a pre-tested structured questionnaire and a follow-up checklist. The main outcomes and causes of death were set by pediatricians and medical residents. EpiData 3.1 and Statistical Package for Social Sciences Version 25 software were used for data entry and analysis, respectively. Multivariable logistic regression was used to identify the predictors of facility-based neonatal mortality.

**Results:**

The proportion of facility-based neonatal mortality was 20% (95% CI:16.7–23.8%). The causes of death were complications of preterm birth (28.58%), birth asphyxia (22.45%), neonatal infection (18.36%), meconium aspiration syndrome (9.18%), respiratory distress syndrome (7.14%), and congenital malformation (4.08%). Low birth weight, preterm births, length of stay of the neonatal intensive care unit, low 5 min APGAR score, hyperthermia, and initiation of feeding were predictors of neonatal death among infants admitted to the neonatal intensive care units of public hospitals in eastern Ethiopia.

**Conclusions:**

The proportion of facility-based neonatal deaths was unacceptably high. The main causes of death were preventable and treatable. Hence, improving the timing and quality of antenatal care is essential for early detection, anticipating high-risk newborns, and timely interventions. Furthermore, early initiation of feeding and better referral linkage to tertiary health facilities could lead to a reduction in neonatal death in this setting.

## Background

The first month is the most crucial period for child survival. Globally, an estimated 2.5 million newborns die in the first month of life, approximately 7000 every day in 2017. Currently, an estimated 18 neonatal deaths per 1000 live births occur during the neonatal period [1, 2], accounting for 46% of under-five deaths [2, 3]. More than 70% of these early neonatal deaths were due to conditions that could be prevented or treated with access to simple and affordable interventions [1, 2, 4–8].

Moreover, 98% of neonatal deaths occur in developing countries with a greater burden occurring in Sub-Saharan Africa (SSA). In this region, approximately one million infant deaths occur in the first month of life, which represents the highest neonatal mortality rate (NMR) among those countries participating in the Sustainable Development Goals (SDGs) and shows the least progress in reducing the NMR [1, 3, 6, 9, 10]. The majority of these deaths are caused by infectious diseases, intrapartum asphyxia, pregnancy-related complications, and premature births [11].

Neonatal death continues to remain a significant public health issue in Ethiopia, with only a slight reduction from 39 per 1000 live births in 2008 to 29 per 1000 live births in 2017 [12, 13]. Facility-based studies in Ethiopia indicated that the proportion of neonatal deaths was 14.3% in Gondar and 23.3% in Addis Ababa [13, 14]. Despite multiple efforts by both the government and other stakeholders, neonatal death remains higher in Ethiopia than in many developing countries [10]. Although the country is committed to achieving SDGs related to both maternal and child health, which was reflected in the Health sector development program IV and the Health sector transformation program, the benefit has not been achieved to date through a reduction in neonatal deaths. The Minister of Health is working collaboratively with the World Health Organization (WHO) to improve the quality of Neonatal Intensive Care Units (NICU) in health facilities; however, more is needed [14, 15].

The continued reduction in neonatal deaths is critical to progress towards achieving SDGs. Therefore, determining facilities-based causes and predictors of neonatal death in the Eastern Ethiopian context is crucial and essential. Due to the recent establishment of the NICU in this setting, data are limited in both the country and study setting. Hence, this study aimed to fill in these gaps and used them as inputs for policymakers and program implementers to design appropriate interventions that could contribute to the reduction of neonatal morbidity and mortality in Ethiopia.

## Methods

### Study area, design, and population

This facility-based prospective follow-up study was conducted among all neonates admitted to the NICU in eastern Ethiopia from November 1 to December 30, 2018. All public health institutions with the NICU facilities in the Harari regional state, Dire Dawa administration and eastern and western Hararghe Zone from the Oromia regional state were included. These facilities have been providing health services for an estimated 6,565,406 populations in the catchment area. A total of 10 public hospitals in this study area provide NICU services. All neonates admitted to the NICU during the study period were included from six randomly selected public hospitals. However, neonates who were admitted for observation and without mothers or guardians during the study period were excluded.

### Sample size and sampling technique

Even though we included all neonates consecutively admitted to the NICU, we checked the adequacy of the sample size using the formula for a double population proportion to determine the sample size using stat calc of Epi Info statistical software Version 7. A 95% confidence level, power of 80%, and an assumption of the ratio of unexposed to exposed equivalent to 1 was assumed for the calculation. Based on this, the sample size was calculated to be 468**.** An addition of 5% was made for the non-response rate, and then the final sample size was 491.

Ten public hospitals in eastern Ethiopia provide NICU services. Among these, six were selected using a simple random sampling technique: Garamuleta, Deder, Chiro, and Sabin Hospital, Dilchora Referral Hospital, and Hiwot Fana Specialized University Hospital were included in the study. Proportional allocation to size was used to distribute the calculated sample size based on the estimated case flow per month in each selected public hospital.

### Data collection methods

A structured questionnaire and checklist adapted from a review of similar literature [4, 6, 16] were used for data collection. The tool contained information related to obstetric and antenatal care (ANC) visits, age at admission, sex, gestational age at birth, APGAR score, admission diagnosis, birth weight, duration of hospital stay, need for ventilator support, neonatal outcomes, and causes of death. It was prepared in English and translated into local languages and then re-translated back into English to ensure consistency and understandability.

Maternal and demographic data were obtained by interviewing the mothers or reviewing the referral records by neonatal nurses, and each neonate was monitored daily until discharged or died. A final assessment of the cause of death was determined by a pediatrician or pediatric resident after conducting the necessary laboratory investigation and a thorough review of medical records.

Prematurity was defined as a live-birth newborn delivered before 37 completed weeks. For participants with an unknown last normal menstrual period, residents used ultrasound and the new Ballard score to estimate the gestational age. Birth weight was classified using WHO weight classification and was measured upon admission using the digital infant weight scale (Seca) [17]. Birth asphyxia was diagnosed when the newborn had at least one of the following signs: not breathing or gasping, < 30 breaths per minute, or < 7 APGAR score, had neonatal neurologic sequelae (seizures, coma, and hypotonia), or multiple organ involvement (kidney, lungs, liver, heart, and intestines) [16, 18].

### Data quality management

A two-day intensive training was given to 12 neonatal nurses, pediatric residents, and pediatricians before data collection. The training consisted of the purpose of the study, enumeration procedures, and how to interview and follow-up the neonates. The tool was pre-tested, and necessary modifications were made for the local context before data collection. After data collection, all completed questionnaires were checked for completeness and cleaned manually. Double data entry was performed to check clarity.

### Statistical analysis

The data were coded and entered into EpiData Version 3.1 and exported to the Statistical Package for Social Sciences (SPSS) Version 25 statistical software for analysis. Univariate analysis was used to describe the frequency distribution of each variable. The outcome variables were coded as “1” for died whereas “0” for improved. The association between the outcome variables (i.e., neonatal death) and independent variables was analyzed using a logistic regression model. Covariates with a *P*-value< 0.2 were retained and entered into the multivariable logistic regression analysis using a forward stepwise approach. Hosmer and Lemeshow’s goodness-of-fit test was used to assess whether the necessary assumptions were fulfilled. Adjusted odds ratio (AOR) with 95% confidence intervals (CI) using a P-value< 0.05 was considered as statistically significant association with the outcome variable.

## Results

### Maternal socio-demographic and ANC characteristics

A total of 489 neonates were admitted during the study period. Two hundred seventy-nine (57.1%) mothers of the neonates were aged 21–30 years with a mean age (±SD) of 26.44 ± 6.04 years. One hundred ninety-eight (40.5%) and 452 (92.4%) mothers were unable to read or write and married. Two hundred and fifty-five (52.1%) participants were from urban residents. Regarding ANC follow-up, two-thirds of mothers 378 (77.33%) had attended at least one prenatal visit; however, only 116 (23.7%) had received the recommended ANC follow-up for their current pregnancy (which was 4 or more ANC visits). One hundred eighty (36.8%) mothers started ANC follow-up before 24 weeks of gestation and 149 (30.5%) started after 24–30 weeks of gestation (Table 1).

**Table 1:** Characteristics of mothers whose neonates were admitted to the NICU of public hospitals in eastern Ethiopia, 2018 [*n* = 489]

Characteristics	Category	Frequency	Percent
Age	less than 20 years	109	22.3
	21–30 years	279	57.1
	31–40 years	95	19.4
	≥ 41 years	6	1.2
Education level	Unable to read and write	198	40.5
	Able to read and write	59	12.1
	Primary Education	111	22.7
	Secondary Education	80	16.4
	College and above	41	8.4
Marital status	Single	28	5.7
	Married	452	92.4
	Divorced	7	1.4
	Widowed	2	0.4
Residence	Urban	255	52.1
	Rural	234	47.9
ANC follow-up	Yes	378	77.3
	No	111	22.7
Place of ANC follow up	Public	320	65.4
	Private	58	11.9
Gestational age at the start of ANC follow up	< 24 weeks	180	36.8
	24–29 weeks	149	30.5
	30–35 weeks	37	7.6
	greater than 35 weeks	12	2.5
Number of ANC follow up attended	One	37	7.6
	Two	80	16.4
	Three	145	29.7
	four and above	116	23.7

### Obstetric characteristics

Two hundred eighty-three (57.9%) neonates were delivered from multiparous mothers. Four hundred forty (90%) were singleton and 380 (77.7%) were delivered at the index hospital. Two hundred ninety-nine (61.1%) mothers were delivered by midwives, and 288 (58.9%) mothers had a labor duration ranging from 4 to 12 hrs. More than two-thirds 338 (69.1%) of mothers were delivered through spontaneous vaginal delivery and 319 (65%) neonates were full-term at birth (Table 2).

**Table 2:** Obstetric characteristics of mothers whose neonates were admitted to the NICU of public hospitals in eastern Ethiopia, 2018 [*n* = 489]

Characteristics	Category	Frequency	Percent
Parity	Primipara	206	42.1
	Multipara	283	57.9
Type of pregnancy	Single	440	90.0
	Twins	49	10.0
Place of delivery	At the index Hospital	380	77.7
	Another hospital	30	6.1
	Health center	53	10.8
	Private facilities	4	0.8
	Home	22	4.5
Birth attendant	Midwife	299	61.1
	Physician	151	30.9
	Nurse	11	2.2
	Health extension workers	2	0.4
	Traditional birth attendants	23	4.7
	Others	3	0.6
Duration of labor	< 4 hrs.	43	8.8
	4-12 hrs.	288	58.9
	Greater than12 hrs.	158	32.3
Amniotic fluid status during labor	Clear	360	73.6
	Meconium stained	107	21.9
	Bloodstained	22	4.5
Mode of delivery	Spontaneous Vaginal	338	69.1
	Cesarean section	122	24.9
	Instrumental Delivery	29	5.9
Gestational age at birth	Very preterm(< 34 weeks)	63	12.9
	Late preterm(34-36 weeks)	97	19.8
	Term(37-42 weeks)	319	65.2
	post-term(> 42 weeks)	10	2.0
Length of hospital stay	< 3 days	160	32.7
	4-7 days	229	46.8
	> 7 days	100	20.4

### Complications during pregnancy

Eighty-one (16.6%) mothers had complications during the index case. Preeclampsia or eclampsia accounted for 43 (8.8%). Besides, 30 (6.1%) mothers had a medical illness during pregnancy. Two hundred twenty-six (46.2%) mothers had complications during labor, with the most common complication being the prolonged duration of labor in 103 (21.1%) (Table 3).

**Table 3:** Obstetric and labor complications among mothers whose neonates were admitted to the NICU of public hospitals in eastern Ethiopia, 2018 [*n* = 489]

Characteristics	Category	Frequency	Percent
Obstetric complications during the current pregnancy	Yes	81	16.6
	No	408	83.4
	Preeclampsia or eclampsia	43	8.8
	Chorioamnionitis	8	1.6
	Premature rupture of membrane	18	3.7
	Antepartum hemorrhage	17	3.5
	Other	1	0.2
Medical illness during pregnancy	Yes	30	6.1
	No	459	93.9
	Diabetes mellitus	5	1.0
	Hypertension	8	1.6
	Tuberculosis	1	0.2
	Cardiac disease	1	0.2
	Anemia	14	2.9
	Human immunodeficiency virus	3	0.6
	Others	2	0.4
Complication during labor	Yes	226	46.2
	No	263	53.8
	Prolonged labor	103	21.1
	Premature rupture of membrane	62	12.7
	Fetal distress	84	17.2
	Cord prolapsed	8	1.6

### Neonatal characteristics

Three hundred six (62.6%) and 378 (77.3%) newborns were male and admitted within the first day of life, respectively. More than half 274 (56.4%) of the neonates had a normal birth weight (2500-3999 g). Three-fourths neonates, 350 (71.6%) were hypothermic upon admission, and 133 (27.2%) suffered from birth asphyxia at 5 min. (based on the 5 min. APGAR score). Three hundred one (61.1%) newborns initiated exclusive breastfeeding in the first hour (Table 4).

**Table 4:** Characteristics of neonates admitted to the NICU of public hospitals in eastern Ethiopia, 2018 [*n* = 489]

Variable	Category	Frequency	Percent
Sex of the newborn	Male	306	62.6
	Female	183	37.4
Age of neonate on admission	≤ one day	378	77.3
	>one days	111	22.7
Birth weight	< 1000 g	8	1.6
	1000–1499 g	33	6.7
	1500–2499 g	145	29.7
	2500–3999 g	274	56.0
	≥ 4000 g	29	5.9
Temperature at admission(^0^c)	< 36.5	350	71.6
	36.5–37.5	92	18.8
	> 37.5	47	9.6
APGAR score at 5 min	≤3	7	1.4
	4–6	126	25.8
	7–10	251	51.3
	Unknown	105	21.5
Initiation of Feeding in 1st hour with	Nothing per mouth	152	31.1
	Exclusive Breast Feeding	301	61.1
	Formula Feeding	36	7.4

### Treatment modalities

**Fig. 1:**
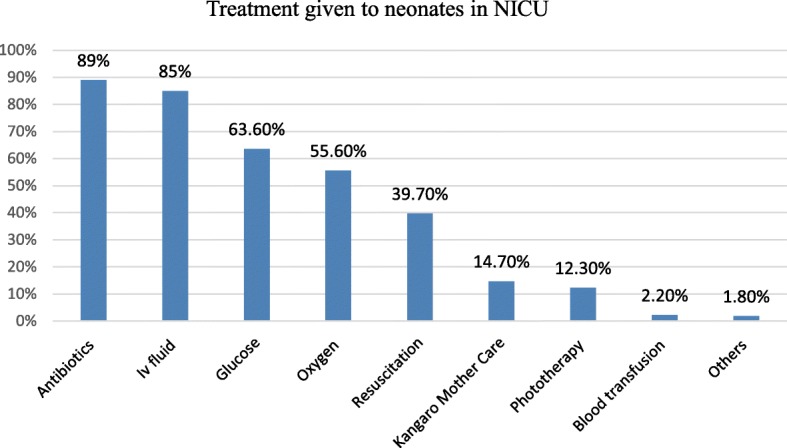
Treatment modalities delivered to neonates admitted to the NICU in public hospitals in eastern Ethiopia, 2018[*n* = 489]

Four hundred thirty-five (89%) neonates had received antibiotics and 416 (85.1%) had intravenous fluids as treatment, while only 72 (14.7%) utilized the kangaroo mother care (Fig. 1).

### Proportion and causes of neonatal mortality

**Fig. 2:**
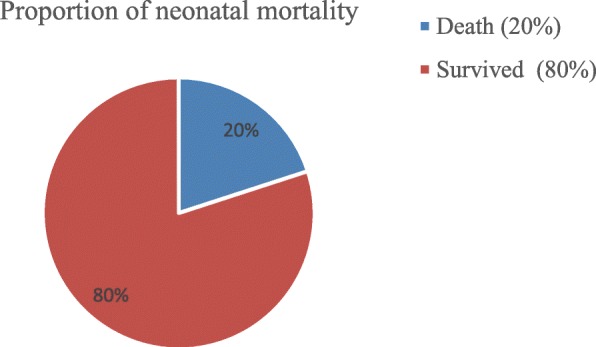
Proportion of neonatal death among neonates admitted to the NICU of public hospitals in eastern Ethiopia, 2018 [n = 489]

**Fig. 3:**
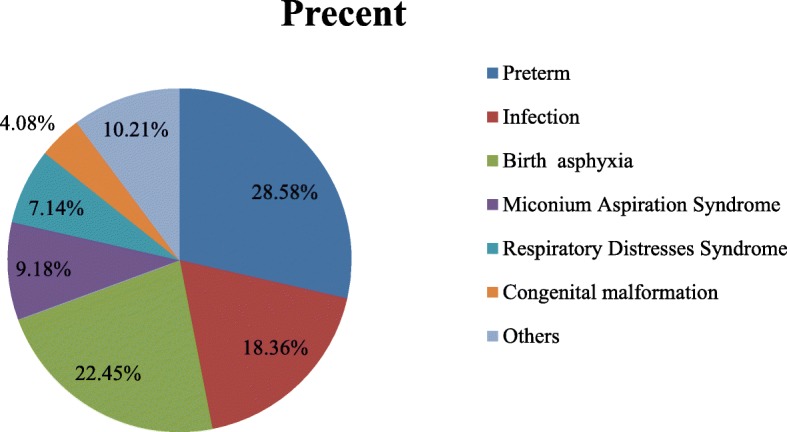
Causes of neonatal mortality among neonates admitted to the NICU of public hospitals in eastern Ethiopia 2018 [*n* = 98]

Among admitted newborns, 98 (20%) died and the remaining survived from admission to discharge in the NICU (Fig. 2). The most common causes of neonatal mortality were complications of preterm 28 (28.58%), followed by birth asphyxia 22 (22.45%) and neonatal infection 18 (18.36%) (Fig. 3).

### Predictors of neonatal mortality in neonatal intensive care units

In the multivariable logistic regression analysis, low 5 min. APGAR score, low birth weight, preterm birth, length of hospital stay, febrile illness, and feeding status were independent predictors of neonatal mortality. Preterm neonates were almost three times (AOR = 2.78; 95% CI: 1.17, 6.57) more likely to die compared with term neonates. Low birth weight babies were two times (AOR = 2.39; 95% CI: 1.04, 5.41) more likely to die when compared with normal birth weight. Furthermore, newborns with a low 5 min. APGAR score were five times(AOR = 5.18;95% CI:2.51,10.66) more likely to die compared with 5 min. APGAR score of greater than or equal to seven; length of stay was another variable found to be predictors of neonatal mortality. Newborns who were hospitalized for less than 3 days were nearly four times (AOR = 3.63; 95% CI: 1.82, 7.22) more likely to die than those who were hospitalized for 4–7 days; this implies that the majority of deaths occurred in the first 72 hrs. of life. Besides, neonates with fever on admission were nearly seven times (AOR = 6.68; 95% CI: 1.34, 33.13) more likely to die compared with their counterparts. Moreover, neonates for whom breastfeeding was not initiated within the first 24 hrs. were twelve times (AOR = 12.16; 95% CI: 5.98, 24.70) more likely to die than those who were on exclusive breastfeeding within the first 24 h of life (Table 5).

**Table 5:** Multivariable analysis for predictors of neonatal mortality among neonates admitted to the NICU of public hospitals in eastern Ethiopia, 2018 [*n* = 489]

Neonatal mortality					
Characteristics	Died	Improved	COR	AOR	*P*-value
Maternal Age					
21–30 years	51	228	1		
< 20 years	29	80	1.62(0.96, 2.73)	1.50(0.67, 3.34)	.335
31–40 years	17	78	0.97(0.53, 1.78)	1.85(0.76, 4.48)	.212
> 40 years	1	5	0.89(0.10, 7.82)	2.40(0.23, 24.89)	.532
Marital status					
Married	88	364	1	1	
Single	9	19	1.96(0.86, 4.48)	1.33(0.35, 5.03)	.766
Others	1	8	0.52(0.06, 4.18)	1.62(0.15, 16.94)	.543
Parity					
Multi-Para	50	233	1	1	
Primi-para	48	158	1.42(0.91, 2.21)	1.94(0.93, 4.05)	.680
Place of delivery					
Inborn (in this hospital)	253	127	1	1	
Other facility	46	41	0.56(0.29, 1.09)	0.50(0.17, 1.51)	.275
Home	16	6	0.35(0.08, 1.53)	0.26(0.03, 2.40)	.205
Duration of labor					
4–12 hrs.	177	111	1	1	
less than 4 hrs.	27	16	0.80(0.35, 1.81)	0.45(0.12, 1.63)	.225
More than 12 hrs.	111	47	0.69(0.41, 1.14)	0.62(0.30, 1.25)	.184
Mode of delivery					
Spontaneous vaginal	221	117	1	1	
Cesarean section	79	43	1.23(0.73, 2.05)	1.00(0.47, 2.10)	.460
Instrumental	15	14	2.78(1.24, 6.17)	1.34(0.40, 4.44)	.944
Length of hospital stay					
4–7 days	172	57	1	1	
< 3 days	68	92	4.03(2.43, 6.67)	3.63(1.82, 7.22) ^*^	.000
> 7 days	75	25	0.77(0.35, 1.63)	0.61(0.23, 1.58)	.310
Gestational age at birth					
Term (37-42 weeks)	227	92	1	1	
Preterm (< 37 weeks)	82	78	3.56(2.23,5.66)	2.78(1.17, 6.56) ^*^	.020
Post term (> 42 weeks)	6	4	4.65(1.25,17.19)	4.54(0.83, 24.70)	.051
Birth weight					
2500–3999 g	211	86	1	1	
Less than 2499 g	77	86	3.46(2.17, 5.49)	2.39(1.04, 5.49) ^*^	.040
≥ 4000 g	27	2	0.23(0.03, 1.73)	0.67(0.06, 6.80)	.858
5 min. APGAR score					
7–10	188	63	1	1	
≤ 6	68	65	5.62(3.32, 9.47)	5.18(2.51, 10.66) ^*^	.000
Unrecorded	59	46	1.33(0.67, 2.60)	2.50(0.82, 7.54)	.092
Temperature on admission					
36.5–37.5c^0^	64	28	1	1	
< 36.5c^0^	218	132	3.37(1.56, 7.23)	2.75(0.97, 7.78)	.057
> 37.5c^0^	33	14	1.25(0.38, 4.05)	6.68(1.35, 33.13) ^*^	.020
Initiation of Feeding					
EBF	237	64	1	1	
NPO	53	99	16.71(9.32, 29.94)	12.16(5.98, 24.70) ^*^	.000
FF	25	11	2.69(0.93, 7.80)	1.71(0.47, 6.15)	.077

*statistically significant at *P* ≤ 0.05; *EBF* exclusive breastfeeding, *NPO* nothing per month, *FF* formula feeding

## Discussion

Despite advancements in technologies and interventions made for improving the life of newborns, neonatal death remains an unfinished agenda as a serious public health concern in developing countries, including Ethiopia. Therefore, we conducted this prospective study to determine the causes and predictors of neonatal mortality in the NICUs in eastern Ethiopia. Accordingly, the proportion of neonatal deaths was 20% (95% CI: 16.7–23.8%). This resulted mainly from preventable causes, which are unconscionable in the twenty-first century. The present finding is in agreement with studies conducted in Ethiopia and other developing countries such as India (18.69%), Eastern Nepal (20.2%), Nigeria (18.8%), and Central Ethiopia (23.3%) [16, 19–21]. However, it was greater than the studies carried out in Northern Vietnam (13.9%), India (7.16%), Ghana (16%), South Africa (13.8%), and Cameroon (15.7%) [5, 22–25]. These variations between studies might be explained, in part, by the discrepancy in the follow-up time, sample size, and quality of prenatal care delivered in these facilities. Also, the present study was conducted in hospitals with a recently established NICU; this might result in a difference in the distribution of essential equipment and skilled manpower. Additionally, disparities in socioeconomic conditions of the population well as geographical locations could have contributed to the variation.

In the current study, the main causes of death were complications of preterm birth (28.58%), birth asphyxia (22.45%), and neonatal infection (18.36%). This was supported by reports and findings from the WHO, Northern Vietnam, India, Ghana, Nigeria, Cameroon, South Sudan, and Southern and Northern Ethiopia [5, 11, 19, 20, 22–24, 26–30]. The findings of our study showed that more than 70% of neonatal deaths were attributed to prematurity, neonatal sepsis, and birth asphyxia. This finding suggests the need for intensive neonatal survival interventions targeting the intrapartum period as well as in immediate and early neonatal periods to reduce neonatal mortality.

Birth asphyxia, preterm, low birth weight, and failure to initiate early feeding were significant factors that increased the likelihood of neonatal deaths. These findings are consistent with previous studies in Ethiopia and other countries, in which intrapartum and neonatal conditions were found to be important predictors of neonatal mortality [21, 25, 28, 30–33]. Preterm and low birth weight babies were more likely to be prone to complications such as hypothermia, infections, and birth asphyxia (resulting in tissue hypoxia and multi-organ failure). Therefore, provisions of quality neonatal care, including quality resuscitation, thermal care, and appropriate feeding, are important to avert some of these factors [34–37].

Furthermore, a short duration of less than 3 days of life in the NICU was found to be significantly associated with neonatal death. This is in contrast with a study in the Somali region, where a shorter stay in the NICU was protective against mortality [38]. However, this is consistent with the fact that most neonatal deaths occur in the first 72 h after birth. This suggests that any intervention at this critical time has a significant contribution to saving the life of the neonates [38, 39]. Besides, febrile illness (≥37.5 ^o^c) on admission was found to be a significant predictor of neonatal mortality. This is likely due to the high proportion of neonatal infections in the present study setting.

This finding emphasizes the need to improve the quality of care in health facilities. In particular, we strongly believe that achieving high-quality intrapartum and postnatal care is required to improve neonatal health. A significant proportion of neonatal deaths can be avoided by appropriate resuscitation care [40, 41]. Most of the infection-related deaths can be avoided by treating maternal infections during pregnancy, ensuring a clean birth, care of the umbilical cord, and exclusive breastfeeding [42, 43]. There is a range of available evidence-based interventions that can improve the survival of premature and low-birth-weight newborns. The promotion of early and exclusive breastfeeding, prevention, and treatment of hypothermia, including kangaroo mother care [43, 44], and topical skin-cleansing with chlorhexidine may reduce morbidity and mortality secondary to infection [45, 46]. The gap for the care of mothers and babies in the first few days of life is important even when women deliver in facilities. Moreover, communities and decision-makers need to be informed that neonatal deaths are a huge portion of child deaths, and need therefore to receive adequate attention.

The strengths of this study were that the inclusion of multiple facilities and prospective follow-up of neonates from admission to discharge or death. In addition, it was carried out without sampling; therefore, the possibility of sampling error was eliminated. However, the relatively small sample size and wide CI in the multivariable model associated with some variables may undermine the strength of this study. The study was conducted at hospitals; therefore, neonates delivered at home and died at home could be missed. Hence, this study does not reflect population-based neonatal mortality rather; it reflects institution-based neonatal death in the given period.

## Conclusion

The proportion of facilities-based neonatal mortality was unacceptably high. The common causes of neonatal death were preterm birth, birth asphyxia, infection, respiratory morbidity, and congenital malformation. Preterm births, low birth weight, low 5 min. APGAR score, short stay in the NICU, failure to initiate early breastfeeding, and temperature (≥37.5 °C) at admission were significant predictors of neonatal mortality in eastern Ethiopia. Efforts to address birth asphyxia, neonatal infections, and preterm birth are critical to achieving survival goals in newborns. Improving the timing and quality of ANC is essential for early detection, anticipating high-risk newborns, and timely interventions. Furthermore, early initiation of feeding and a better referral linkage to tertiary facilities could contribute to the reduction of neonatal mortality and morbidity in this setting.

## Data Availability

All the data of this study are available from the corresponding author upon reasonable request.

## Abbreviations

ANC: Antenatal Care
AOR: Adjusted Odd Ratio
APGAR: Appearance, Pulse, Grimace, Activities, and Respiration
CI: Confidence Interval
CSA: Central Statistical Agency
EBF: Exclusive Breast Feeding
MAS: Meconium Aspiration Syndrome
NICU: Neonatal Intensive Care Unit
NMR: Neonatal Mortality Rate
RDS: Respiratory Distress Syndrome
SDGs: Sustainable Development Goals
SPSS: Statistical Package for Social Sciences
UNICEF: United Nations International Children’s Emergency Fund
WHO: World Health Organization
